# COVID-19 Booster Vaccination Hesitancy in the United States: A Multi-Theory-Model (MTM)-Based National Assessment

**DOI:** 10.3390/vaccines10050758

**Published:** 2022-05-11

**Authors:** Kavita Batra, Manoj Sharma, Chia-Liang Dai, Jagdish Khubchandani

**Affiliations:** 1 Trauma and Critical Care, Department of Medical Education, Kirk Kerkorian School of Medicine, University of Nevada, Las Vegas, NV 89102, USA; 2 Department of Social and Behavioral Health, School of Public Health, University of Nevada, Las Vegas, NV 89119, USA; manoj.sharma@unlv.edu; 3 Department of Teaching and Learning, College of Education, University of Nevada, Las Vegas, NV 89154, USA; chia-liang.dai@unlv.edu; 4 Department of Public Health Sciences, New Mexico State University, Las Cruces, NM 88003, USA; jagdish@nmsu.edu

**Keywords:** SARS-CoV-2, COVID-19, vaccine booster, vaccine literacy, vaccine confidence

## Abstract

**Background**: Despite the availability of COVID-19 vaccines and the proven benefits of vaccinations outweighing the potential risks, hesitancy to accept vaccines and additional doses remains a persistent problem. Therefore, the purpose of the study was to investigate hesitancy, confidence, literacy, and the role of the multi-theory model (MTM) constructs in COVID-19 booster uptake. **Methods**: This cross-sectional study utilized a 52-item psychometric valid web-based survey conducted during the month of October 2021 to recruit a nationally representative sample of U.S. adults. Univariate, bivariate, and multivariate statistical tests were used to analyze the data. **Results:** Among the booster hesitant group (*n* = 209, 41.7%), a significantly larger proportion of respondents were unvaccinated with the primary series (43.5% vs. 11%, *p* < 0.001), were among 18–44 years age group (51.2% vs. 31.8%, *p* < 0.001), single or never married (33.0% vs. 24.3%, *p* = 0.04), had lower education with some high school (6.2% vs. 2.4%, *p* = 0.03), and identified themselves as Republicans (31.6% vs. 20.5%, *p* = 0.01). The hesitant group had lower mean scores of vaccine literacy, and vaccine confidence, and had 19% lower odds of behavioral confidence than their non-hesitant counterparts (adjusted odds ratio = 0.81, 95% CI: 0.71–0.92). **Conclusions**: The findings of this study underscore the need of raising public awareness through effective multi-theory-model-based communication campaigns.

## 1. Introduction

By April 2022, globally, there were more than 500 million cases of COVID-19 infection and more than six million deaths due to COVID-19 [1]. By this time, more than 80 million were infected in the United States alone, and 1 million people died due to COVID-19 [2]. The ongoing COVID-19 pandemic remains concerning as the virus that caused COVID-19 has changed over time, along with the documentation of reinfection with COVID-19 among individuals [3]. As the COVID-19 variant emerges and the pandemic continues, vaccination remains one of the most effective strategies in disease prevention and public health promotion as vaccines reduce the severity of illness and death from COVID-19 [4,5]. Moreover, findings from several studies indicate that full vaccination and booster doses provide additional protection against reinfection. This is particularly important, as the reinfection of COVID-19 is not uncommon [6,7]. Cavanaugh and colleagues (2021) reported that among previously infected individuals, those who were not vaccinated were twice as likely to be re-infected compared to those who were fully vaccinated [8]. Another study reported that those who were unvaccinated had 2.6 and 5.3 times, respectively, higher rates of incidence and hospitalization due to COVID-19 compared with those who were fully vaccinated [9]. In addition, Levine-Tiefenbrun et al. (2021) examined the COVID-19 vaccine’s effect on the viral load among positive cases who were vaccinated. The researchers found that the viral load was significantly lower among vaccinated people compared with unvaccinated people [10].

Researchers also have found that a booster dose of the vaccine provides effective protection against COVID-19-related symptoms and hospitalization [11]. Spitzer and colleagues (2022) examined the association between the booster dose and the infection rate of COVID-19 among 1928 health care workers [12]. Findings of the study indicated that among participants who previously completed full vaccination, those who received a booster dose compared with those not receiving one showed a significantly lower rate of COVID-19 infection. Bar-on et al. (2021) also found that rates of COVID-19 infection and related severe illness were significantly lower among participants who received a booster dose of vaccine compared with those who did not [4]. There is robust evidence now to show that vaccination and booster doses for COVID-19 help reduce infections and poor outcomes among infected individuals.

Despite the availability of COVID-19 vaccines and the proven benefits of vaccinations outweighing the potential risks, hesitancy to accept vaccines remains a persistent global problem [13,14]. People choose not to receive vaccines due to a variety of reasons, including but not limited to concerns about the vaccine’s side effects or its effectiveness, misinformation, mistrust of experts and authorities, lack of knowledge or fear, etc. [15,16]. Volpp and colleagues (2021) argued that strategies with a focus on addressing facts and evidence about COVID-19 vaccines and establishing vaccine confidence and acceptance in all populations are key to increasing vaccine rates [17].

Theory-based interventions in promoting behavioral health provide information regarding what factors contribute to targeted preventive health behaviors [18]. The multi-theory model (MTM) of health behavior change was designed to address both initiation and sustenance of health behavior change [19]. This theory integrates cognitive, conative, and environmental factors that are intended to be utilized for designing health behavior change interventions. As the virus continues to mutate and evolve, it is of paramount importance to investigate the public’s perceptions toward the COVID-19 vaccine booster shots. In addition, the utilization of theory-based approaches is critical for the development of evidence-based interventions to increase COVID-19 vaccine uptake. Thus far, there are limited theory-based studies conducted to understand the COVID-19 vaccine booster shot hesitancy. Therefore, the purpose of the study was to investigate hesitancy, confidence, literacy, and the role of MTM constructs in COVID-19 booster dose uptake. The results of the study will help in developing effective COVID-19 vaccine promotion programs.

## 2. Materials and Methods

### 2.1. Data Collection

This cross-sectional study utilized a web-based survey conducted during the month of October 2021 to recruit a nationally representative sample of U.S. adults. The data collection for this study was performed as a part of commercial services offered by Qualtrics, which utilizes the market research panels through specialized campaigns [20,21]. Several invitation methods, including emails, and in-app notifications were used by the Qualtrics market research team to collect the data for this study. The eligibility of the participants was assessed by a few screening questions posed at the beginning of the survey to limit any self-selection bias. Specific details of the study were provided once participants identified themselves as eligible for the study. Upon completion of the survey, respondents were compensated for their time as per agreement with their Panel Providers in the form of SkyMiles, gift certificates, cash, etc.

The sample was predetermined using the formula: *n* = (z)^2^ *p* (1 − *p*)/d^2^ with a 95% confidence interval (alpha = 0.05, z = 1.96), a margin of error d = 5%, and the proportion of booster dose hesitancy among Americans was 38% based on the data reported by Yadete et al., in December 2021 [22]. The estimated sample size was 399 (363 + 10% non-response = 399) after accounting for 10% non-response. The sample of the current study was relatively larger than the predetermined sample size, which was sufficient to see the hypothesized effects.

### 2.2. Ethical Considerations

The study was conducted according to the guidelines of the Declaration of Helsinki and approved by the Institutional Review Board (or Ethics Committee) of the University of Nevada, Las Vegas (UNLV-2021-108 dated 4 October 2021). Detailed information about the study’s objectives, procedures, expected outcomes, and risk was provided to the participants so they could make informed decisions about their participation. Participants were also informed that participation was voluntary and that they could withdraw from the study at any time.

### 2.3. Study Measures

The survey instrument used in this study consisted of 52 items related to vaccine confidence [23], vaccine literacy [24], multilevel-theory-model-based initiation of vaccination behavior [25,26], and demographic questions. Vaccine literacy instruments (14 items) include functional (5 items), iterative or communicative (5 items), and critical literacy (4 items). Functional literacy (which includes questions about language capabilities and about the semantic system) was measured on a Likert scale ranging from “4—never” to “1—often.” Iterative or communicative and critical literacy (which are related to cognitive efforts and problem-solving) were measured on the same scale but with a reverse scoring (“1—never”, and “4—often” criteria [23]. All the scores of subtypes of literacy were combined to calculate total vaccine literacy. Vaccine Confidence Index was calculated on the basis of 8 Likert-type statements with answer choices related to agreement or disagreement: “1—totally agree”, “2—partially agree”, “3—partially disagree”, and “4—totally disagree” [24].

The MTM-based initiation was measured through subscales, including perceived advantages, perceived disadvantages, behavioral confidence, and changes in the physical environment [19]. Perceived advantages (3 items) and perceived disadvantages (3 items) scores were measured on a 5-point Likert scale ranging from “never” to “very often.” The difference between the summative scores of perceived advantages and perceived disadvantages was termed participatory dialogue. The remaining two constructs, i.e., behavioral confidence (3 items) and changes in the physical environment (3 items) were measured on a surety scale ranging from “Not at all sure” to “Completely sure”. The possible score range for MTM constructs was 0–12 units. The MTM tool is based on the fourth-generation behavioral theory and has a long-standing history to measure a variety of behaviors amidst the COVID-19 pandemic [27,28,29].

### 2.4. Data Analysis

**Figure 1:**
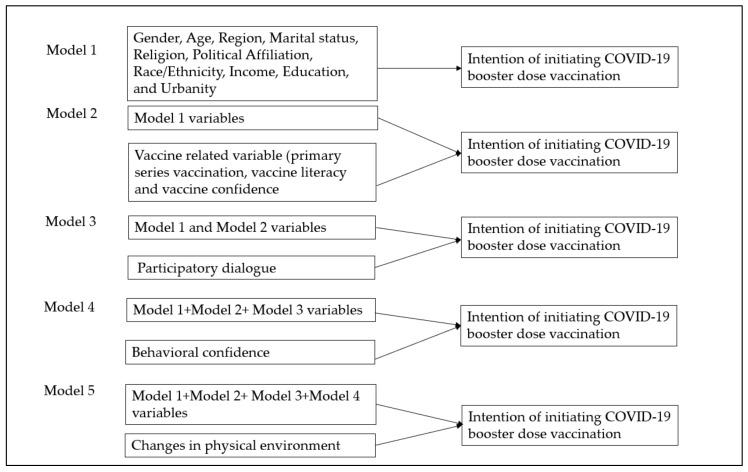
Hierarchical regression model building process.

Data were first cleaned and recoded for the analytical operations. All statistical assumptions, including the normality, homogeneity of variance, independence of residuals, and equal error variances, were assessed. Box plots were visually inspected to identify outliers in the data. Continuous variables are described as mean and standard deviation unless stated otherwise. Categorical variables were represented as frequencies and proportions. Univariate and bivariate analyses (i.e., chi-square, independent-samples *t*-test, and Pearson’s correlation test) were used to describe the sample. In univariate statistics, 95% confidence intervals of proportion were calculated using normal approximation to the binomial distribution. Adjusted standardized residuals greater than 2 were considered significant cells for contingency tables larger than 2 × 2 chi-square analysis. A posthoc contingency table analysis using adjusted residuals (or Z scores) was performed to generate *p* values of multiple comparisons. Hierarchical multiple regression was run to determine if the addition of primary vaccination status, vaccine literacy, vaccine confidence, and MTM-based subscales’ participatory dialogue, behavioral confidence, and changes in the physical environment improved the prediction of initiating booster dose acceptability above demographic variables alone (Figure 1, model building process). A multivariable logistic regression model was fit to generate adjusted odds ratios for booster dose hesitancy. Estimates of parameters were obtained through the maximum likelihood estimation method with 95% Wald’s confidence limits for the logistic model. The final model was selected based upon the Akaike Information Criterion (A1C) and the Schwarz Criterion (SC) [30]. For regression analyses, polytomous categorical variables were dummy-coded to calculate accurate parameters. All tests were two-sided, and a *p*-value of < 0.05 was considered significant. The Statistical Package for Social Sciences for Windows, version 27.0 (SPSS, Chicago, IL, USA) and Statistical Analysis System (SAS 9.4) were used to analyze the data.

## 3. Results

A total of 501 respondents completed the survey where the majority (75.4%) of the respondents were either fully vaccinated with the primary series or not hesitant toward the booster dose (58.3%) (Table 1). The mean age of the participants was 51.21 (SD: 18.8) years with nearly 40% of respondents being in the 18–44 years age group. A little less than half of the participants were males or married (Table 1). The majority (>50%) of the participants were White, had less than a college degree, had annual incomes less than USD 50,000, or selected Christianity as their religion (Table 1).

Among the booster-hesitant group (*n* = 209, 41.7%), a significantly larger proportion of respondents were unvaccinated with the primary series (43.5% vs. 11%, *p* < 0.001), were among the 18–44 years’ age group (51.2% vs. 31.8%, *p* < 0.001), single or never married (33.0% vs. 24.3%, *p* = 0.04), had a lower level of education with some high school (6.2% vs. 2.4%, *p* = 0.03), and identified themselves as Republicans (31.6% vs. 20.5%, *p* = 0.01, Table 2).

The results in Table 3 indicate the mean differences in vaccine literacy and confidence among booster-hesitant and non-hesitant groups. Compared to the booster non-hesitant group, the hesitant group had statistically significantly lower mean scores for functional literacy (15.82 ± 3.50 vs. 12.80 ± 3.99) iterative or communicative literacy (15.45 ± 3.25 vs. 14.71 ± 3.26, *p* = 0.02), critical literacy (13.10 ± 2.73 vs. 12.0 ± 2.81, *p* < 0.001), total vaccine literacy (44.36 ± 6.71 vs. 39.62 ± 6.42), and vaccine confidence index (2.77 ± 1.14 vs. 1.07 ± 0.55, *p* < 0.001) (Table 3).

As indicated in Table 4, the mean scores of MTM initiation and its subscales, including perceived advantages, behavioral confidence, and changes in the physical environment were higher among the non-hesitant group. On the contrary, the mean scores of “perceived disadvantages” were higher among the booster hesitant group (7.81 ± 2.77 vs. 4.36 ± 2.61), with a statistically significant difference of 3.44 (95% CI: 2.96, 3.92, t (499) = 14.192, *p* < 0.001, d = 1.30 (Table 4).

**Figure 2:**
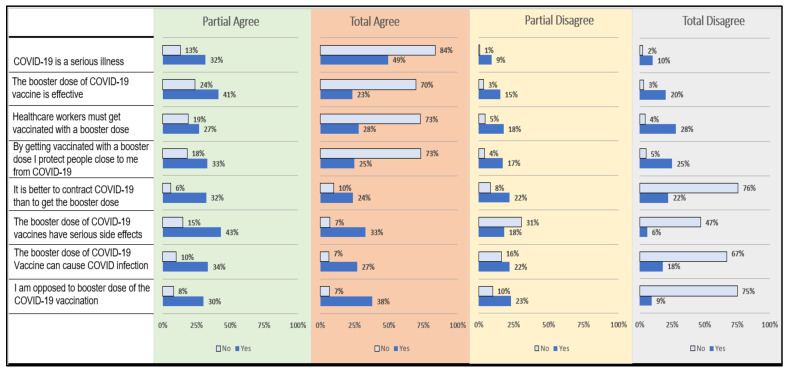
Item-wise comparison of vaccine confidence index among booster dose hesitant (yes) and non-hesitant (no) groups. Note: All differences were statistically significant.

Upon item-wise analysis of vaccine confidence, a significantly larger proportion of the non-hesitant group believed in the effectiveness and protective role of the booster dose as compared to the booster hesitant group. In contrast, booster-hesitant respondents were opposed to the booster dose and thought that it can have side effects in addition to the secondary COVID-19 infection (Figure 2). All differences in proportions were statistically significant.

Bivariate correlations (Table 5) indicated that perceived advantages are inversely correlated with perceived disadvantages (r = −0.40, *p* < 0.01) and directly correlated with behavioral confidence (r = 0.68, *p* < 0.01), changes in physical environment (r = 0.64, *p* < 0.01), age (r = 0.09, *p* < 0.05), vaccine literacy (r = 0.35 *p* < 0.01), and vaccine confidence (r = 0.65, *p* < 0.01). Behavioral confidence was directly correlated with the changes in physical environment (r = 0.81, *p* < 0.001), vaccine literacy (r = 0.42, *p* < 0.001), and vaccine confidence (r = 0.64, *p* < 0.001). The Cronbach alpha values of the entire scale and only the MTM scale were 0.83 and 0.82, respectively. The perceived disadvantages were inversely correlated with the vaccine confidence (r = −0.63, *p* < 0.01) and vaccine literacy (r = −0.26, *p* < 0.01). Vaccine confidence and vaccine literacy were also positively correlated with each other (r = 0.48, *p* < 0.001, Table 5).

In relation to the hierarchical regression model (Table 6), the full model of demographic and vaccine-related variables and MTM subscales to predict initiation of booster dose vaccination behavior (Model 5) was statistically significant, R^2^ = 0.684, F (30, 172) = 12.411, *p* < 0.001; adjusted R^2^ = 0.629. The addition of primary series vaccination status, vaccine literacy, and vaccine confidence to the prediction of booster dose vaccination behavior (Model 2) led to a statistically significant increase in R^2^ of 0.200, F (3, 175) = 20.605, *p* < 0.001. The addition of participatory dialogue to the prediction of booster dose vaccination behavior (Model 3) also led to a statistically significant increase in R^2^ of 0.063, F (1, 174) = 21.785, *p* < 0.001. The addition of behavioral confidence to the prediction of booster dose vaccination behavior (Model 4) also led to a statistically significant increase in R^2^ of 0.174, F (1, 173) = 91.327, *p* < 0.001.

**Figure 3:**
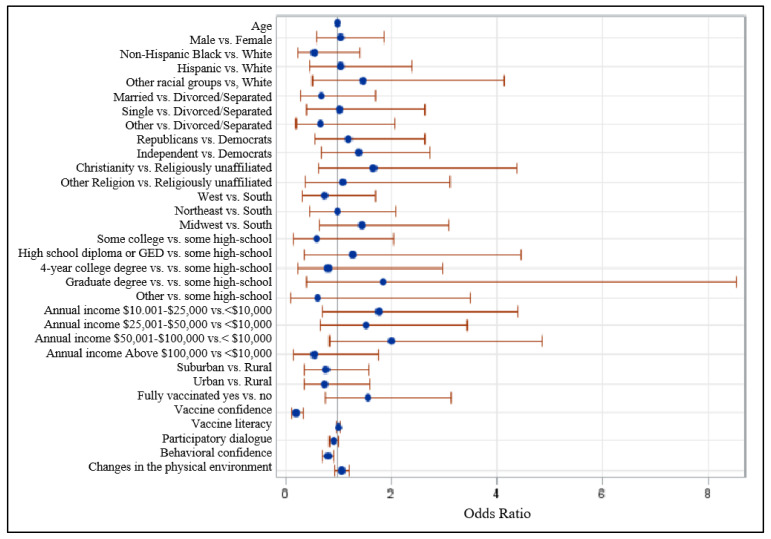
Forest plot showing odds rations and 95% Wald confidence intervals. Note: Probability modeled in booster hesitancy = 1.

As shown in the Forest plot graph (Figure 3), after adjusting for all confounders, the booster-hesitant group had 79% lower odds of having vaccine confidence as compared to the non-hesitant group (adjusted odds ratio = 0.21, 95% CI: 0.125–0.351). Likewise, the booster hesitant group had 19% lower odds of having behavioral confidence than their non-hesitant counterparts (adjusted odds ratio = 0.81, 95% CI: 0.71–0.92). 

## 4. Discussion

Booster doses for COVID-19 vaccines in the US were approved in early Fall 2021, and by April 2022, more than 95 million American adults received a booster dose [31,32]. This would mean that the majority of fully vaccinated adults have not received a booster dose by April 2022. Additionally, the results from our study suggest that the majority (>50%) of fully vaccinated; Americans are hesitant toward receiving a booster dose. Given the emergence of new COVID-19 variants (e.g., Omicron variant, BA2 variant after the approval of booster doses), dwindling immunity from prior vaccination, and the long gap between primary vaccination and approval for boosters, effective interventions are needed to increase the uptake of booster doses in the American population. The results of our study indicate critical avenues for interventions to increase the uptake of booster doses in the US population and among other countries where boosters have recently been approved or might be approved soon [33].

Three notable findings of this study need greater consideration to understand COVID-19 vaccination hesitancy and to design appropriate interventions. First, as it relates to sociodemographic characteristics, younger and never married, African Americans, those with lower education, and Republicans were the most hesitant toward the booster dose. While these findings are disconcerting due to the high risk of COVID-19 infection and poor outcomes associated with the infection among some of these groups, they are not entirely surprising. Even before the COVID-19 vaccines were rolled out and also during the active rollout phase, some of these groups expressed hesitancy toward the COVID-19 vaccination calling for greater targeted and tailored vaccine-related communication for these groups [26,34,35]. Second, as it relates to vaccine confidence and literacy, those with lower confidence and literacy were more likely to express hesitancy toward a booster dose. Again, these findings call for greater communication with the public about the safety profile and utility of a booster dose. For example, a recent analysis of more than 80 million Americans who received a booster dose (by investigators from the CDC and FDA) found that serious side effects with a booster were rare, and in fact, side effects were less common with boosters than with the second primary series dose [36]. Studies of special population groups (e.g., healthcare workers) and from other countries have also found an association between vaccine confidence and booster dose willingness [37,38]. Third, and finally, the MTM-related constructs were found to be strong predictors of booster dose vaccination behavior. Behavioral confidence or the sureness to take the vaccine booster was found to be a strong predictor. In general terms for relevance around the world, this construct can be viewed as the level of confidence an individual has to receive a COVID-19 booster. This construct can be built through educational interventions. Likewise, the construct of changes in the physical environment was found to be a significant predictor. In general terms, for relevance around the world, this construct can be viewed as the availability, accessibility, and obtainability of a COVID-19 booster vaccine. This construct can be influenced by both policies and the availability of boosters. This finding mirrors the results from the plethora of studies on vaccine hesitancy conducted before the rollout of COVID-19 vaccines and during the rollout of the primary series of vaccines [22,25,26,39,40,41]. This indicates a need for sustained and ongoing culturally relevant communication regarding the benefits of these vaccines and their usefulness along with efforts to reduce barriers and perceived disadvantages of these vaccines and their booster doses. Regarding booster doses, vaccine and pandemic fatigue, side effects from prior doses, breakthrough infections, and lower perceived risk of the disease with declining cases of infections could lead to questioning by the public about the utility and need of the boosters, their effectiveness, and whether boosters are a viable solution for pandemic control [41,42,43].

Given the three notable findings of this study, evidence-based interventions based on effective communication strategies are warranted for communities. Until there is enough vaccination around the world to hinder the emergence of newer COVID-19 variants, in countries where boosters have been approved and vaccines are available, the public should be educated about the ongoing risk and severity of infections with new variants. Additionally, the public should be informed about the large-scale successful trials of booster doses and their efficacy and safety. In the published literature, most recommendations to increase uptake of COVID-19 vaccinations focus on primary series vaccination. However, results from our study indicate that these recommended strategies can be utilized with some changes based on the local context. For example, a popular model (C’s model for an increase in vaccination) suggests that local leaders, healthcare providers, and the mass media should emphasize the reduction in Complacency and Constraints, increase Confidence and Calculations in favor of vaccines, and promote Collective responsibility by Communication for a unique local Context with Comparative analyses of the risks of having COVID-19 infection versus COVID-19 vaccination. Such communication about booster doses should also consider the findings of our study and emphasize addressing public concerns (e.g., of safety and side effects of vaccines) and increasing awareness about the availability and need for booster doses [22,36,41,42,43]. Our study found that MTM can be a useful framework for designing such educational communication campaigns. Potential advantages of boosters should be underscored, behavioral confidence should be built through highlighting multifarious sources of confidence, and advertisements about the accessibility of boosters should be part of the booster acceptance campaigns.

### Strengths and Limitations

The results of this study should be viewed in light of several potential limitations. First, our findings are restricted by all threats to the validity and reliability inherent to cross-sectional and survey study designs (e.g., socially desirable responses, non-response bias, self-selection bias, recall bias, and the inability to establish cause-and-effect relationships). Second, although the MTM is a comprehensive model, there could be other individual characteristics and influential factors that could have influenced study participants’ willingness to receive a booster dose for COVID-19 (e.g., side effects from previous doses of the vaccine, mandates from employers, or COVID-19 related mortality and morbidity in social networks) [36,39]. Third, our study sample had a higher proportion of individuals who were vaccinated with the primary series of the COVID-19 vaccine (75% in our study vs. 65% of the US population). Finally, a threat to the external validity is that the sample is limited in nature and extent (e.g., limited to those with computers or mobile phones and an understanding of the online survey environment). Despite these limitations, our study on COVID-19 vaccine booster dose hesitancy is among the few studies in the US, which utilized a theory-based survey tool. Most of the earlier studies on booster dose hesitancy are either from outside the US or focus on unique populations such as healthcare workers. Additionally, the majority (>50%) of our sample consisted of adult Americans who were Whites, females, non-Hispanic, employed full-time, urban or suburban residents, living in the South or West, with less than a college degree, and with annual household income less than USD 50,000. These numbers closely resemble the US population distribution as per the Census making our study sample highly representative of the US population to a great extent [41,42,43]. 

## 5. Conclusions

This study aimed to examine the role of hesitancy, confidence, literacy, and MTM constructs in COVID-19 booster dose uptake. The study showed that more than half of fully vaccinated Americans were hesitant toward receiving a booster dose of the COVID-19 vaccine. In terms of designing concerted communication interventions, it was found that it would be useful to target campaigns for younger, never married, African Americans, those with lower education, and Republican political affiliation. The communication interventions need to focus on building vaccine confidence and literacy. This can be achieved by designing interventions that are based on the multi-theory model (MTM) of health behavior change that operationalizes the constructs of participatory dialogue in which advantages of booster acceptance are highlighted over putative disadvantages, and behavioral confidence is built in a stepwise manner through role modeling and exploring sources of confidence, and changes in the physical environment being developed by initiating robust policies and making the boosters easily accessible and informing the public about them.

## Figures and Tables

**Table 1:** Demographic characteristics of the respondents (*N* = 501).

Variable Name	Categories	*n* (%)	95% CI (LCL, UCL) of Proportion
Vaccinated status (Primary Series)	Yes	378 (75.4)	71.4, 79.2
	No	123 (24.6)	20.8, 28.5
Hesitancy toward COVID-19 booster dose	Yes	209 (41.7)	37.4, 46.2
	No	292 (58.3)	53.8, 62.6
Age groups	18–44 years	200 (39.9)	35.6, 44.4
	45–64 years	145 (28.9)	25.0, 33.1
	65 years or older	156 (31.1)	27.1, 35.4
Gender	Male	237 (47.3)	42.8, 51.7
	Female	255 (50.9)	46.4, 55.3
Race/ethnicity	Non-Hispanic White	291 (58.1)	53.6, 62.4
	Non-Hispanic African American	66 (13.2)	10.3, 16.5
	Hispanic	96 (19.2)	15.8, 22.8
	Other (including multiracial groups)	48 (9.5)	7.1, 12.5
Marital status	Divorced/Separated	69 (13.8)	10.8, 17.1
	Other	56 (11.2)	8.4, 13.9
	Single, never married	140 (27.9)	24.1, 32.1
	Married	236 (47.1)	42.6, 51.5
Education	High school diploma or GED	125 (25.0)	21.2, 28.9
	4-year college degree	126 (25.1)	21.4, 29.2
	Graduate-level degree	55 (11.0)	8.4, 14.1
	Some college	158 (31.5)	27.5, 35.8
	Some high school	20 (4.0)	2.5, 6.1
	Other, including vocational training schools	17 (3.4)	1.9, 5.4
Income	USD 10,000 or below	34 (6.8)	4.7, 9.4
	USD 10,001–USD 25,000	79 (15.8)	12.6, 19.2
	USD 25,001–USD 50,000	139 (27.7)	23.8, 31.8
	USD 50,001–USD 100,000	131 (26.1)	22.4, 30.2
	Above USD 100,001	49 (9.8)	7.3, 12.7
Pre-existing conditions	Yes	158 (31.5)	27.5, 35.8
	No	343 (68.5)	64.2, 72.5
Region	Midwest	97 (19.4)	15.9, 23.1
	Northeast	102 (20.4)	16.9, 24.2
	South	218 (43.5)	39.1, 47.9
	West	84 (16.8)	13.6, 20.3
Urbanity	Rural	134 (26.7)	22.9, 30.8
	Suburban	211 (42.1)	37.7, 46.5
	Urban	156 (31.1)	27.1, 35.4
Political affiliation	Democrat	181 (36.1)	31.9, 40.5
	Republican	126 (25.1)	21.4, 29.2
	Independent	145 (28.9)	25.0, 33.1
	Others	49 (9.8)	7.3, 12.7
Religion	Christianity	304 (60.7)	56.2, 64.9
	Religiously unaffiliated	63 (12.6)	9.8, 15.8
	Others	134 (26.7)	22.9, 30.8

Note: Some percentages may not add up to 100% as a few respondents preferred not to answer. Other religions include Hinduism, Judaism, Buddhism, Islam, etc.

**Table 2:** Bivariate comparison of Booster Dose hesitancy by sample characteristics (*N* = 501).

Variable Name	Categories	Booster Hesitant		*p*-Value	Statistics	ES
		Yes (*n* = 209, 41.7%)	No (*n* = 292, 58.3%)			
Vaccinated status (Primary Series)	Yes	118 (56.5)	260 (89.0)	**<0.001**	69.810	0.373
	No	91 (43.5)	32 (11.0)			
Age groups	18–44 years	107 (51.2)	93 (31.8)	**<0.001**	21.589	0.208
	45–64 years	56 (26.8)	89 (30.5)	0.4		
	65 years or older	46 (22.0)	110 (37.7)	**<0.001**		
Gender	Male	93 (44.5)	144 (49.3)	0.4	3.079	0.078
	Female	111 (53.1)	144 (49.3)			
	Other *	NR	NR			
Race/ethnicity	Non-Hispanic White	127 (60.8)	164 (56.2)	0.3	4.143	0.091
	Non-Hispanic African American	20 (9.6)	46 (15.8)	0.05 **		
	Hispanic	42 (20.1)	54 (18.5)	0.7		
	Other (including multiracial groups)	20 (9.6)	28 (9.6)	0.9		
Marital status	Divorced/Separated	29 (13.9)	40 (13.7)	0.9	5.099	0.101
	Married	91 (43.5)	145 (49.7)	0.2		
	Other	20 (9.6)	36 (12.3)	0.2		
	Single, never married	69 (33.0)	71 (24.3)	**0.04**		
Education	High school diploma or GED	60 (28.7)	65 (22.3)	0.1	9.156	0.135
	4-year college degree	46 (22.0)	80 (27.4)	0.2		
	Graduate-level degree	19 (9.1)	36 (12.3)	0.3		
	Some college	64 (30.6)	94 (32.2)	0.7		
	Some high school	13 (6.2)	7 (2.4)	**0.03**		
	Other	7 (3.3)	10 (3.4)	0.9		
Income	USD 10,000 or below	17 (9.3)	17(6.8)	0.4	6.448	0.122
	USD 10,001–USD 25,000	39 (21.3)	40 (16.1)	0.2		
	USD 25,001–USD 50,000	60 (32.8)	79 (31.7)	0.8		
	USD 50,001–USD 100,000	53 (29.0)	78 (31.3)	0.6		
	Above USD 100,001	14 (7.7)	35 (14.1)	**0.04**		
Pre-existing conditions	Yes	67 (32.1)	91 (31.2)	0.8	0.045	0.009
	No	142 (67.9)	201 (68.8)			
Region	Midwest	44 (21.1)	53 (18.2)	0.4	6.079	0.110
	Northeast	38 (18.2)	64 (21.9)	0.3		
	South	100 (47.8)	118 (40.4)	0.09		
	West	27 (12.9)	57 (19.5)	0.05 **		
Political affiliation	Democrat	50 (23.9)	131 (44.9)	**<0.001**	24.015	0.219
	Republican	66 (31.6)	60 (20.5)	**0.01**		
	Independent	71 (34.0)	74 (25.3)	**0.04**		
	Others	22 (10.5)	27 (9.2)	0.6		
Religion	Christianity	133 (63.6)	171 (58.6)	0.3	1.324	0.051
	Religiously unaffiliated	24 (11.5)	39 (13.4)	0.4		
	Others	52 (24.9)	82 (28.1)	0.4		
Urbanity	Rural	63 (30.1)	71 (24.3)	0.1	2.285	0.068
	Suburban	82 (39.2)	129 (44.2)	0.2		
	Urban	64 (30.6)	92 (31.5)	0.8		

*p* values are Bonferroni corrected for multiple comparisons; ES: effect size; * not reported due to *n* < 5. The percentage may not add to 100% as some respondents preferred not to report some demographics; ** marginally significant; *p* values less than 0.05 are considered statistically significant and are bolded in the table.

**Table 3:** Vaccine literacy and confidence among booster dose hesitant and non-hesitant groups (*N* = 501).

Variable Name	Booster Dose Hesitancy		*p*-Value	Test Statistics	Effect Size
	Yes (*n* = 209)	No (*n* = 292)	-		
Functional literacy	12.80 ± 3.99	15.82 ± 3.50	<0.001	−8.930	0.80 [Large]
Communicative literacy	14.71 ± 3.26	15.45 ± 3.25	0.02	−2.487	0.23 [Small]
Critical literacy	12.0 ± 2.81	13.10 ± 2.73	<0.001	−4.000	0.40 [Small]
Total vaccine literacy	39.62 ± 6.42	44.36 ± 6.71	<0.001	−7.940	0.72 [Large]
Vaccine Confidence Index	1.07 ± 0.55	2.77 ± 1.14	<0.001	−21.974	1.80 [Very large]

Note: All measures are represented as Mean ± standard deviation unless stated otherwise.

**Table 4:** MTM initiation and its subscale scores among COVID-19 booster dose hesitant and non-hesitant group (*N* = 501).

MTM Construct	Booster Dose Hesitancy		*p*-Value	Test Statistics	Effect Size
	Yes (*n* = 209)	No (*n* = 292)	-		
Overall Initiation Score	1.35 ± 1.39	3.27 ± 1.08	<0.001	−16.624	1.50 [Very large]
Subscales					
Perceived Advantages	5.56 ± 3.43	8.66 ± 2.60	<0.001	−10.989	1.04 [Large]
Perceived Disadvantages	7.81 ± 2.77	4.36 ± 2.61	<0.001	14.192	1.30 [Very large]
Behavior Confidence	4.49 ± 3.79	9.03 ± 3.10	<0.001	−14.694	1.33 [Very large]
Changes in the Physical Environment	5.57 ± 4.09	9.37 ± 2.98	<0.001	−11.429	1.10 [Large]

Note: All measures are represented as Mean ± standard deviation unless stated otherwise.

**Table 5:** Pearson correlations, and reliability estimates for study variables in the sample population (*n* = 501).

Variables	1	2	3	4	5	6	7
1. Perceived Advantages	1	−0.403 **	0.683 **	0.636 **	0.098 *	0.351 **	0.651 **
2. Perceived Disadvantages	−0.403 **	1	−0.462 **	−0.376 **	−0.160 **	−0.262 **	−0.631 **
3. Behavioral Confidence	0.683 **	−0.462 **	1	0.811 **	0.115 *	0.422 **	0.644 **
4. Change in the Physical Environment	0.636 **	−0.376 **	0.811 **	1	0.199 **	0.471 **	0.592 **
5. Age	0.098 *	−0.160 **	0.115 *	0.199 **	1	0.155 **	0.302 **
6. Total literacy score	0.351 **	−0.262 **	0.422 **	0.471 **	0.155 **	1	0.481 **
7. Vaccine Confidence	0.651 **	−0.631 **	0.644 **	0.592 **	0.302 **	0.481 **	1
*Cronbach’s Alpha*	0.92	0.78	0.93	0.93	-	0.80	0.80

** *p* < 0.01; * *p* < 0.05.

**Table 6:** Hierarchical multiple regression (HRM) predicting the intention of COVID-19 booster dose acceptability among hesitant respondents (*n* = 209).

Variables	Model 1		Model 2		Model 3		Model 4		Model 5	
	B	*β*	B	*β*	B	*β*	B	*β*	B	*β*
Constant	1.821 *	-	−0.087	-	0.391	-	−0.089	-	0.175	-
Age	−0.012	−0.125	−0.011	−0.144	−0.007	−0.095	0.001	0.007	−0.001	−0.014
Gender (Ref: Female)	0.102	0.037	0.181	0.065	0.167	0.060	0.117	0.042	0.088	0.031
Non-Hispanic African American (Ref: White)	0.174	0.037	0.143	0.031	0.225	0.048	0.004	0.001	−0.029	−0.006
Hispanic	0.361	0.105	0.260	0.076	0.365	0.107	0.215	0.063	0.236	0.069
Other	0.139	0.028	−0.051	−0.01	−0.097	−0.02	−0.068	−0.014	−0.05	−0.01
USD 10.001–USD 25,000 (Ref: Income under USD 10,000)	0.81 *	0.225	0.42	0.118	0.34	0.094	0.207	0.058	0.2	0.056
USD 25,001–USD 50,000	0.42	0.138	0.27	0.090	0.24	0.077	0.111	0.036	0.132	0.043
USD 50,001–USD 100,000	0.33	0.103	0.12	0.039	0.21	0.065	0.012	0.004	0.019	0.006
Above USD 100,000	0.70	0.128	0.35	0.064	0.44	0.080	0.047	0.009	0.024	0.004
Married (Ref: Divorced/Separated)	−0.098	−0.035	−0.175	−0.063	−0.184	−0.066	0.114	0.041	0.153	0.055
Single	−0.063	−0.021	−0.016	−0.005	−0.05	−0.017	0.169	0.057	0.205	0.069
Other	0.125	0.026	0.155	0.033	0.215	0.045	0.339	0.071	0.349	0.073
Suburban (Ref: Rural)	0.285	0.100	0.241	0.085	0.210	0.074	0.220	0.077	0.137	0.048
Urban	0.413	0.138	0.336	0.112	0.274	0.091	0.244	0.081	0.197	0.066
Republicans (Ref: Democrats)	−0.547 *	−0.184	−0.276	−0.093	−0.177	−0.060	−0.246	−0.083	−0.262	−0.088
Other, including independent	−0.872 **	−0.312	−0.570 *	−0.204	−0.401	−0.144	−0.406 *	−0.145	−0.431 *	−0.154
Christianity (Ref: Religiously unaffiliated)	0.778 *	0.270	0.610 *	0.212	0.558 *	0.193	0.210	0.073	0.189	0.065
Others	0.430	0.134	0.490	0.152	0.486	0.151	0.140	0.043	−0.433 *	−0.155
West (Ref: South)	0.019	0.004	0.060	0.014	0.001	0.003	0.071	0.017	0.095	0.022
Northeast	0.263	0.074	0.045	0.013	0.006	0.002	0.054	0.015	0.046	0.012
Midwest	−0.049	−0.014	−0.082	−0.024	−0.089	−0.026	−0.027	−0.008	0.004	0.001
Some college (Ref: Some high school)	−0.414	−0.137	−0.314	−0.104	−0.133	−0.044	−0.057	−0.019	−0.051	−0.017
High school diploma or GED	−0.075	−0.025	−0.248	−0.081	−0.149	−0.049	−0.184	−0.058	−0.202	−0.066
4 years of a college degree	−0.342	−0.103	−0.260	−0.079	−0.137	−0.041	−0.266	−0.080	−0.260	−0.078
Graduate	0.292	0.060	−0.023	−0.005	0.011	0.002	−0.114	−0.023	−0.110	−0.023
Others	−0.774	−0.102	−0.732	−0.096	−0.382	−0.050	−0.245	−0.033	−0.184	−0.024
Primarily vaccinated (Ref: No)	-	-	0.999 **	0.359	0.756 **	0.271	0.511 *	0.183	0.420 *	0.150
Vaccine Literacy	-	-	0.024	0.111	0.026 *	0.117	0.001	0.002	−0.008	−0.036
Vaccine Confidence	-	-	0.522 *	0.207	0.039	0.016	0.198	0.079	0.222	0.088
Participatory dialogue	-	-	-	-	0.109 **	0.377	0.037	0.130	0.031	0.108
Behavioral confidence	-	-	-	-	-	-	0.202 **	0.548	0.170 **	0.461
Changes in the physical environment	-	-	-	-	-	-	-	-	0.059 *	0.172
R^2^	0.233	-	0.433	-	0.496	-	0.670	-	0.684	-
F	2.257 *	-	4.958 **	-	6.127 **	-	12.136 **	-	12.411 **	-
Δ R^2^	0.233	-	0.200	-	0.063	-	0.174	-	0.014	-
Δ F	2.257 *	-	20.605 **	-	21.785 **	-	91.327 **	-	7.387 *	

* *p*-value < 0.05; ** *p*-value < 0.001; Adjusted R^2^ of initiation in the final model = 0.629.

## Data Availability

The data presented in this study are available on request from the corresponding author. The data are not publicly available due to ethical reasons.
